# Designing a decision aid for cancer prevention: a qualitative study

**DOI:** 10.1093/fampra/cmad042

**Published:** 2023-04-14

**Authors:** Shakira Milton, Finlay Macrae, Jennifer G McIntosh, Sibel Saya, Pavithran Alphonse, Thivagar Yogaparan, Napin Karnchanachari, Kitty Novy, Peter Nguyen, Phyllis Lau, Jon Emery

**Affiliations:** Centre for Cancer Research, University of Melbourne, Melbourne, Australia; Department of General Practice, University of Melbourne, Melbourne, Australia; Department of Medicine, The University of Melbourne, Melbourne, Australia; Colorectal Medicine and Genetics, The Royal Melbourne Hospital, Melbourne, Australia; Centre for Cancer Research, University of Melbourne, Melbourne, Australia; Department of General Practice, University of Melbourne, Melbourne, Australia; Centre for Cancer Research, University of Melbourne, Melbourne, Australia; Department of General Practice, University of Melbourne, Melbourne, Australia; Centre for Cancer Research, University of Melbourne, Melbourne, Australia; Department of General Practice, University of Melbourne, Melbourne, Australia; Centre for Cancer Research, University of Melbourne, Melbourne, Australia; Department of General Practice, University of Melbourne, Melbourne, Australia; Centre for Cancer Research, University of Melbourne, Melbourne, Australia; Department of General Practice, University of Melbourne, Melbourne, Australia; Centre for Cancer Research, University of Melbourne, Melbourne, Australia; Department of General Practice, University of Melbourne, Melbourne, Australia; Health and Government Sector, Quantium, Melbourne, Australia; Department of General Practice, University of Melbourne, Melbourne, Australia; School of Medicine, University of Western Sydney, Sydney, Australia; Centre for Cancer Research, University of Melbourne, Melbourne, Australia; Department of General Practice, University of Melbourne, Melbourne, Australia

**Keywords:** aspirin, cancer prevention, chemoprevention, colorectal cancer, decision aid, general practice, guideline implementation, primary care

## Abstract

**Objectives:**

Australian guidelines recommend people aged 50–70 years old consider taking low-dose aspirin to reduce their risk of colorectal cancer. The aim was to design sex-specific decision aids (DAs) with clinician and consumer input, including expected frequency trees (EFTs) to communicate the risks and benefits of taking aspirin.

**Methods:**

Semi-structured interviews were conducted with clinicians. Focus groups were conducted with consumers. The interview schedules covered ease of comprehension, design, potential effects on decision-making, and approaches to implementation of the DAs. Thematic analysis was employed; independent coding by 2 researchers was inductive. Themes were developed through consensus between authors.

**Results:**

Sixty-four clinicians were interviewed over 6 months in 2019. Twelve consumers aged 50–70 years participated in two focus groups in February and March 2020. The clinicians agreed that the EFTs would be helpful to facilitate a discussion with patients but suggested including an additional estimate of the effects of aspirin on all-cause mortality. The consumers felt favourable about the DAs and suggested changes to the design and wording to ease comprehension.

**Conclusion:**

DAs were designed to communicate the risks and benefits of low-dose aspirin for disease prevention. The DAs are currently being trialled in general practice to determine their impact on informed decision-making and aspirin uptake.

**Key messages**

- Aspirin reduces the risk of bowel cancer and is recommended in Australia.
- A decision aid was developed with input from 64 clinicians and 12 consumers.
- Clinicians agreed that the decision aid would support a discussion with patients.
- Clinicians improved the expected frequency tree and added mortality data.
- Consumers improved the design and made the decision aids more comprehensible.
- It is being trialled to test whether it supports informed choice and aspirin use.

## Introduction

In Australia, in 2021, there were 15,540 new cases of colorectal cancer, including 8,247 males and 7,239 females.^1^ Colorectal cancer was the third most common cause of cancer death with 5,295 deaths.^2^ Daily low-dose aspirin reduces the incidence of and mortality from colorectal cancer by up to 25% and 33%, respectively.^3^ Cancer Council Australia guidelines state that for Australians aged 50–70 years old who are at average risk of colorectal cancer, aspirin should be actively considered to prevent colorectal cancer. A low dose (100–300 mg per day) of aspirin should be considered for 2.5–5 years to prevent colorectal cancer.^4^ This recommendation is based on level I evidence and categories as a grade B recommendation. Despite this, many clinicians are unaware of the guidelines as there was no active plan for embedding them into clinical practice.^5^ Decision aids (DAs) can increase the uptake of preventive health interventions,^6^ and are a potential method for implementing the aspirin guidelines into clinical practice.

DAs are evidence-based tools designed to facilitate a discussion and encourage shared decision-making between clinicians and patients to support complex healthcare decisions. A systematic review found DAs were beneficial for facilitating discussions between patients and clinicians to improve knowledge, reduce decisional conflict, and encourage patients to take an active role in decision-making.^7^

There is clear guidance available to help researchers and clinicians design DAs.^6–8^ Typically, DAs contain the following: information about the risks and benefits of a particular treatment or intervention, information about the disease, and contraindications or who should not consider the treatment. DAs should be informative but easy to understand for both patients and clinicians.^9^

In our previous work, different graphical methods were tested to communicate the benefits and harms of taking low-dose aspirin to primary care patients aged 50–70 years, including an icon array, expected frequency tree (EFT), bar chat, and government letter of recommendation. It was concluded that an EFT was acceptable and easily understood by consumers.^10^ In additional preliminary research about the Australian aspirin guidelines for colorectal cancer prevention, it was found that a wider implementation strategy focused on primary care was required, including DAs to facilitate discussions between general practitioners (GPs) and patients.^5^

The aim of this study was to design sex-specific DAs, the men benefit more from taking aspirin, and obtain feedback from both clinicians and consumers as end users. The aim was to explore all aspects of the DA, including ease of comprehension, design aspects, potential effects on decision-making, and how to implement the DA into practice.

## Methods and materials

### Forming the steering group and deciding on a decision aid

A steering committee was formed, comprising the authors (*N* = 11), from various professional backgrounds, including an academic general practitioner (GP), a gastroenterologist, cancer researchers, a genetic counsellor, and several research students.^6^ It was decided that the scope of the DA was to present an informed decision-making tool for the Cancer Council Australia aspirin guidelines for colorectal cancer prevention and that the audience would be clinicians and patients.

### Approach

The aim was to develop a DA iteratively through interviews with clinicians and focus groups with consumers, obtaining feedback specifically on the EFT as well as the overall content and format of the DA. A constructivist paradigm where it is believed that people construct their own knowledge and interpretation of the world,^11^ was utilized. Therefore, data from participants reflected different interpretations and meanings they derived from the DA, which refined the EFTs.

### Setting and sampling strategy

#### Clinician participants

Purposive sampling was used to recruit clinicians as part of an overarching qualitative study,^5^ which explored their opinions about using the aspirin guidelines in clinical practice.^5^ Clinicians were recruited through professional networks, social media posts, and snowball sampling. Clinicians were included in they lived and worked in metropolitan or regional Victoria, Australia, and were excluded if they lived in far remote locations as it was intended that we would interview them face to face. We also had a sampling matrix, so intentionally recruited more female clinicians. Presented here are results about clinicians’ understanding of the EFT and the feedback they provided to improve the design and ease of comprehension of it. Most interviews were conducted face to face while a few GP interviews were conducted over the phone.

#### Consumer participants

Consumers were recruited through purposive sampling to ensure participants were diverse in socio-economic status, education, and from both rural and urban Victoria. Our sampling matrix for the focus group participants included only these characteristics. Recruitment was done through the Primary Care Collaborative Cancer Clinical Trials Group’s (PC4) Community Advisory Group, authors’ personal and professional networks, and snowball sampling. Participants were reimbursed for their time with $100 vouchers.

### Data collection techniques

Clinicians participated in one-on-one interviews in their workplace, facilitated by SM, PA, and TY. The portion of the clinician interview schedule for this project can be found in Supplementary File A. The consumers participated in semi-structured focus groups (schedule Supplementary File B). SM led the focus groups, ensuring equal participation from, while NK and KN helped to facilitate the sessions. The focus groups were held in a meeting room within the Victorian Comprehensive Cancer Centre and were audio recorded and transcribed by a professional transcription company.

### Analysis

Data from the clinician interviews and focus groups were organized using NVivo 12.^12^ All clinician interviews were coded by the author who conducted the interviews, and 2 to 3 interviews were coded by another author and additionally independently coded by another author (JM). To enhance interpretive rigour, SM and NK independently fully coded focus group interviews. Independent codes were merged, and similar codes were combined. Using thematic analysis,^13^ similar codes were organized into topics and grouped into overarching themes.

### Development process of expected frequency trees and decision aids

**Fig. 1.**
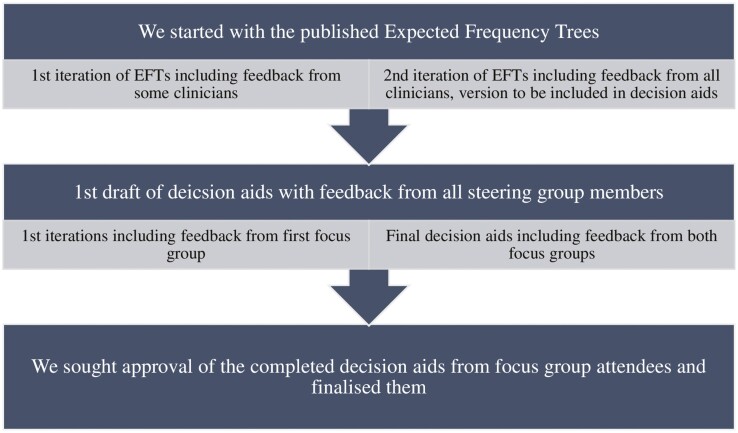
The process of designing a decision aid for cancer prevention, for the “Designing a decision aid for cancer prevention” study.

A visual overview of the EFT and DA development process can be found in Figure 1.

#### Developing EFTs

The original sex-specific EFTs from an earlier study^10^ were refined in this study through an iterative process. As each iteration of the EFTs was developed, the disease incidence estimates were updated, and overall mortality estimates were added. The methods for calculating these estimates for each clinical outcome presented in the EFTs are described in Nguyen et al.^10^ for the original versions and in Supplementary File C for alterations made in this study.

#### Developing decision aids

Separate male and female DAs were designed as the incidence of disease and benefit of taking aspirin differs between the sexes.^14^ The first draft of the DA incorporated a summary statement of the Cancer Council Australia recommendation, the EFTs on the benefits and harms of aspirin from our previous study^10^ contraindications to aspirin, and a reminder to complete colorectal cancer screening.

The DAs, including the EFTs were updated after both focus groups were complete and, after analysis was complete. Final versions of the DAs were posted to all focus group attendees for final approval. They were offered to email us back with further suggestions to improve the DA for the research team.

## Results

Sixty-four clinicians, including familial cancer clinic staff (geneticists, oncologists, and genetic counsellors), gastroenterologists, pharmacists, and GPs were interviewed by 3 researchers (SM, TY, and PA) between March and October 2019 (Table 1). Two focus groups were conducted in February and March 2020 (Table 2).

**Table 1.** Characteristics of clinician participants *n* = 64 in the “Designing a decision aid for cancer prevention” study.

Characteristics	*n* (%)
Mean age (years)	41
Sex, number of women	35 (54.7)
Participants’ profession	
Gastroenterologist	17 (26.6)
Pharmacist	14 (21.9)
General practitioner	16 (25.0)
Familial cancer on (FCC) staff	
Genetic counsellor	10 (15.6)
Geneticists	4 (6.3)
Oncologist	3 (4.6)
Years of professional work experience	
<10	23 (35.9)
10–19	22 (34.4)
20–29	8 (12.5)
30+	11 (17.2)
Work setting for participants	
General practice	
Bulk-billing clinic	20 (31.3)
Private	44 (68.7)
Hospital (gastroenterologists and FCC staff)	
Public	49 (76.5)
Private	16 (23.4)
Pharmacy	
Hospital	23 (35.9)
Community	41 (64.1)

Interviews were conducted in Victoria, Australia between March and October 2019.^5^

**Table 2.** Characteristics of consumer participants (*n* = 12) in the focus groups for the “Designing a decision aid for cancer prevention” study.

Characteristics	%
Age brackets	
40–59	25
60–74	59
Missing	16
Sex	
Female	25
Male	59
Missing	16
Born in Australia	67
Born overseas	16
Missing	16
Ethnicity	
Caucasian/Anglo-European	75
Aboriginal and/or Torres Strait Islander origin	0
Maori/Pacific Islander	0
Asian	9
Missing	16
Education	
Up to TAFE	25
University degree or higher	59
Missing	16
Annual household income	
Up to $49,999	16
$50,000–$79,999	8
$80,000–$124,999	42
$125,000–$199,999	8
$200,000+	0
Prefer not to answer	8
Missing	16
Postcode^15^	
Regional Victoria	42
Metropolitan Victoria	42
Missing	16
Marital status	
Married	66
Living with a partner	-
Single, never married	9
Divorced	9
Missing	16
Currently taking aspirin	
100 mg daily	33
300 mg daily	0

Two focus groups were conducted in Victoria Australia in February and March 2020.

**Fig. 2.**
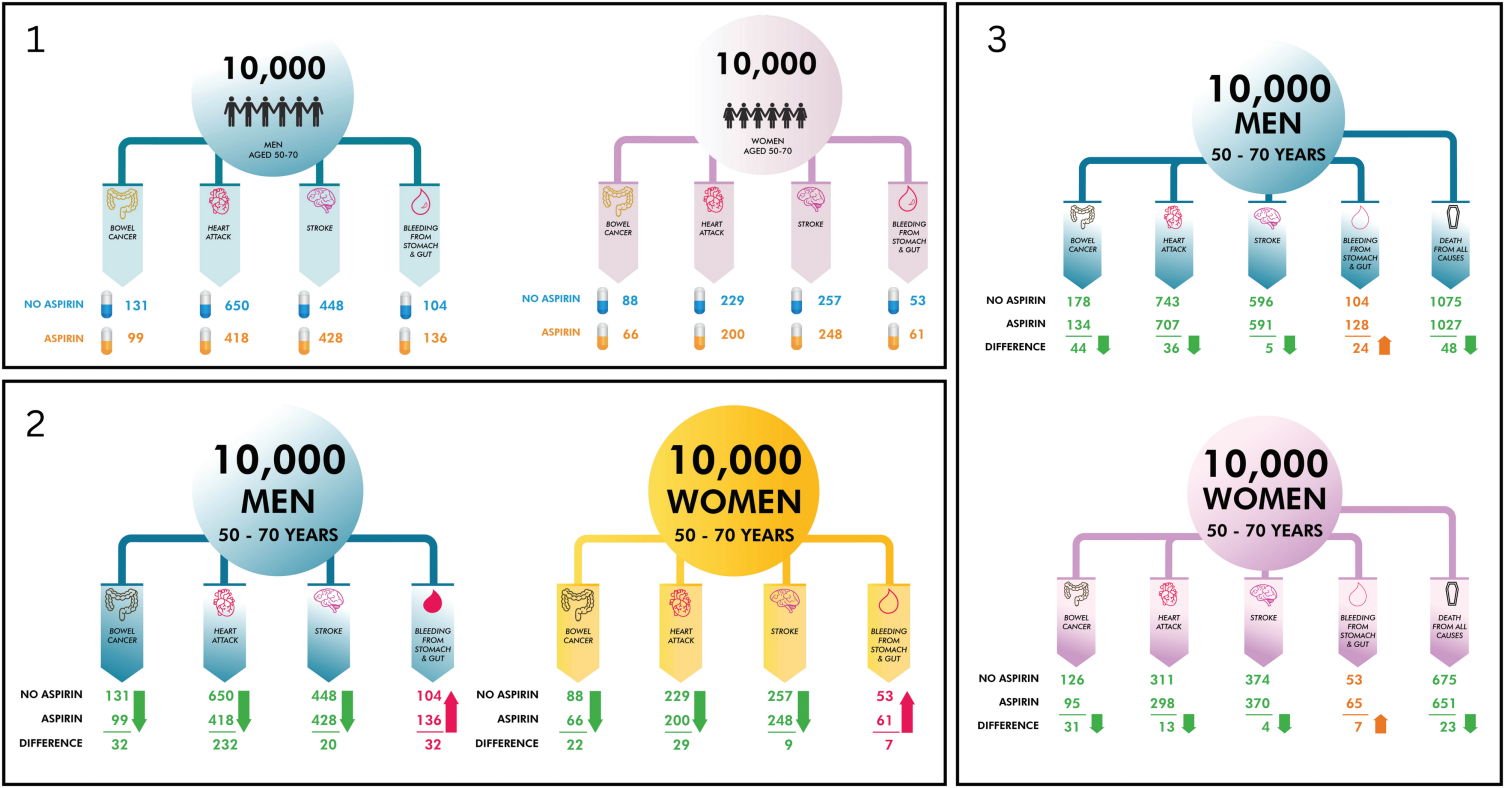
The original expected frequency trees before clinician feedback were published in Emery et al.^16^. 2: First iteration of the expected frequency trees, including some of the initial feedback from clinicians. 3: Final expected frequency trees after clinician feedback, showing the effects of aspirin on the INCIDENCE of events and MORTALITY from all causes over 10 years of taking aspirin for at least 5 years in Australian men and women aged 50–70 years, for the “Designing a decision aid for cancer prevention” study.

The EFT iterations can be found in Figure 2. The DAs iterations can be found in Supplementary Files D–F. Table 3 shows how the data-informed changes to the DAs and the EFTs within it, with supporting quotes presented in Tables 4 and Table 5 for clinicians and consumers, respectively.

**Table 3.** Explanation of how design changes made to the expected frequency trees and decision aids relate to ease of comprehension for the “Designing a decision aid for cancer prevention” study.

DAs or EFTs	Figure/Table/Supplementary file	Quote number	Accompanying quote	Changes made
EFTs	Figure 2	Table 4. Quotation 1	“So, I’m trying to do some maths in my head [laughs]. If I look at a diagram, I don’t really like to look at maths, do some maths” Male, GP, 45 years GP010	Between versions 1 and 2, difference lines were added to make it easier to interpret the expected frequency tree
EFTs	Figure 2	Table 4. Quotation 3	“But I don’t know, I think maybe it would be helpful to have arrows or something next to them. Just to show, so I don’t have to think too much about reading the numbers.” Female, genetic counsellor, FC009	Between versions 1 and 2, we removed the tablet images and added arrows to indicate an increase or decrease in the difference in incidences of bowel cancer, heart attack, stroke, and mortality. Between versions 2 and 3, we moved the arrows next to the difference line to only highlight that the difference either went up or down.
EFTs	Figure 2	Table 4. Quotation 5	“And I think it should be neutral colours” Female, Pharmacist, 32 years “I think the colours and the arrows, and you can see obviously significant benefits from it, yeah I think that looks really good.” Female, GP, 28 years	Between versions 1 and 2, we changed the colour of the female DA from pink to yellow, then we changed it to lilac in version 3 as we received positive feedback on having gendered colours as they are easy to specify that they are for males and females and would limit picking up the wrong one
EFTs	Figure 2	Table 4. Quotation 6	“I guess in all the - like just the recent hot topic of all that sort of intersex and the unisex toilets and things like that, so I guess whilst very - I can understand quickly and pictorially the typical symbol for the women for women’s toilets and men’s toilets would be one of these, but I don’t know, you could just perhaps get away with not having a person there at all and just have women a bit bigger, and men a bit bigger.” Female, genetic counsellor, FC014	We removed the male and female symbols from the DAs as they did not serve a numerical purpose and were originally put on the DAs for design and comprehension
EFTs	Figure 2	Table 4. Quotation 7	“It might be useful to have mortality - overall mortality as well. To say even, these things have improved and as a result, the death rate reduced as well.” Female, Oncologist, FC016	Mortality was added to version 3, the final version of the DAs (Supplementary File B)
Das	E and F to G and H Second iteration to final	Table 5. Quotations 2 and 4	“The other thing I didn’t - this is nothing to do with the brochure - but I don’t understand the instruction that says take Aspirin for two and a half years” Male “Just split that a bit and say, how much Aspirin should I take? So, you’d say 100 to 300 milligrams. Then after two and a half years, the statistics show that you’ll get better.” Male	As the consumers found the dose and duration of use, of aspirin to be confusing, they asked that we split them and change the wording. We added “baby aspirin” and a minimum of 2 ½ to 5 years. We changed 2.5 to 2 ½ as a ½ was easier to comprehend than 0.5
Das	E to G	Table 5. Quotation 9	“So, if I wasn’t reading the words and looking at it going, oh it’s pink, its breast cancer, oh she’s lost her hair.” Female	The image of the female on the second iteration of the DA had short hair, the image combined with the pink colour reminded the consumers of breast cancer awareness, so we changed the image and colour to a lilac
Das	E and F to G and H	Table 5. Quotation 8	“I think it’s a great idea and as I said my main thing would be, I would just be happy if it was just one because I can see I’m the person that would take the wrong one” Female	Consumers suggested that we combine the DAs into one so they would not accidentally take the wrong one, but the steering group decided against the suggestion. To limit males and females from taking the incorrect brochures we added the words male and female to the front in bold font. We also added the age range to target the correct patients.
Das	E and F to G and H	Table 5. Quotations 10 and 11	“Yeah, maybe smaller tablets, yeah.” Male “Considering I don’t take tablets that look like that.” Male	The image of the large aspirin tablet was changed to an image of a blister tablet pack
DAs	E and F to G and H	Table 5. Quotation 12	“Oh, there’s a point, make it big enough that we don’t have to put our glasses on.” Female	We changed the font throughout the DAs to make it larger and increased the font size to make it easier for consumers to read without glasses.

**Table 4.** Clinician quotes during the one-on-one interviews conducted to gain feedback on the design of the expected frequency trees, hypothetical decision making and ideas for implementation in clinical practice, for the “Designing a decision aid for cancer prevention” study.

Number	Clinician quotations
Ease of comprehension and design aspects of EFTs	
1	“So, I’m trying to do some maths in my head [laughs]. If I look at a diagram, I don’t really like to look at maths, do some maths” Male, GP
2	“I find the absolute numbers quite hard to conceptualise. I think percentages might be helpful.” Female, genetic counsellor
3	“But I don’t know, I think maybe it would be helpful to have arrows or something next to them. Just to show, so I don’t have to think too much about reading the numbers.” Female, genetic counsellor
4	“I’d say it’s far too complicated in terms of colour coding. You have the same colour of benefits and complications. And so, it’s hard to work out what’s good and bad.” Gastroenterologist, Male
5	“I think the colours and the arrows, and you can see obviously significant benefits from it, yeah I think that looks really good.” Female, GP
6	“I guess in all the - like just the recent hot topic of all that sort of intersex and the unisex toilets and things like that, so I guess whilst very - I can understand quickly and pictorially the typical symbol for the women for women’s toilets and men’s toilets would be one of these, but I don’t know, you could just perhaps get away with not having a person there at all and just have women a bit bigger, and men a bit bigger.” Female, genetic counsellor
7	“It might be useful to have mortality - overall mortality as well. To say even, these things have improved and as a result, the death rate reduced as well.” Female, Oncologist
Potential effects on decision-making about aspirin	
8	“I think it’s a fabulous idea. It really makes it very clear. The benefits and risks.” Male, Geneticist, 54 years
9	“So, for instance, if now - I will be having more of these conversations around aspirin in terms of colorectal cancer, do they understand what the risks versus the benefits are and using the pictogram, if it’s possible to get a copy of that.” Male, GP
10	“I think it’s a good way of presenting it. It depends on how numerate people are, and we’ve certainly had a lot of experience with patient-facing materials like this, some people like their pictograms of the number of people affected by different things.” Female, Genetic counsellor
11	“This is quite busy. I think even maybe - just for me, everywhere I am at the moment, the health literacy is so low, that I think this will be way too much information for my current demographic.” Female, GP
12	“I think most patients would of course say that when you’re talking about some gastritis versus a diagnosis of bowel cancer, you’re not comparing like with like. So, I think that most people wouldn’t be concerned by that.” Female, Genetic counsellor
13	“Far too complicated. My patients sort of... probably something like 60 per cent of my patients, English is their second language… not many of my patients would find it useful.” Male, Gastroenterologist
14	“In my experience, most of these shared decision-making tools, the big disadvantage of them is the time that they take.” Female, GP
Approaches to implementation	
15	“I think it would be good in the GPs’ rooms or something like that where you’re talking - or the person who’s doing the prescribing or talking a bit more about it might be helpful because they’re going to have to manage the heart attack or the stroke aspect.” Female, Genetic Counsellor
16	“But the RACGP has a decision tree in terms of PSA testing, which is another controversial area for GPs. Then it’s a graphical representation and then it helps patients put their understanding of the risk of PSA testing in perspective.” Male, GP

**Table 5.** Consumer quotes from focus groups were conducted to gain feedback on the design and ease of comprehension of the decision aids, hypothetical decision-making, and ideas for implementation in clinical practice, for the “Designing a decision aid for cancer prevention” study.

Number	Consumer quotations
Ease of comprehension and design aspects	
1	“I don’t understand the instruction that says take Aspirin for two and a half years” Male
2	“It says to me that after two and a half years you might start to see some benefit, but it won’t happen, is that what it says?” Female
3	“Just split that a bit and say, how much Aspirin should I take? So, you’d say 100 to 300 milligrams. Then after two and a half years, the statistics show that you’ll get better.” Male
4	“This is the grammar police in here. It’s like, it says here if you’re between the ages of 50 and 70, just speak to your GP about taking 100 to 300 milligrams of Aspirin every day for at least two and a half years to prevent colorectal cancer. It says for at least two points five years, there’s no, I think that that should be another one where it’s got minimum, instead of at least” Female
5	Well, I was thinking it was more of a medical terminology thing. Someone in layman’s terms, wouldn’t understand it. I work in a hospital, and I’ve never heard [of] it. Female
6	“But if it’s related to stomach ulcers, though, you would think that stomach ulcers would cover the whole thing. So, I reckon it’s probably not necessary to even be on there.” Female
7	“I think it’s a great idea and as I said my main thing would be, I would just be happy if it was just one because I can see I’m the person that would take the wrong one” Female
8	“So, if I wasn’t reading the words and looking at it going, oh it’s pink, it’s [for] breast cancer, oh she’s lost her hair.” Female
9	“Yeah, maybe smaller tablets, yeah.” Male
10	“Considering I don’t take tablets that look like that.” Male
11	“Oh, there’s a point, make it big enough that we don’t have to put our glasses on.” Female
Potential effects on decision-making about aspirin for consumers	
12	“You’re right, some of them [brochures in general practice waiting rooms] are very wordy, aren’t they, in the waiting room?” Female
13	“If I wasn’t taking Aspirin, I would consider taking it for the statistics you’ve got here.” Male
14	“So, it’s making a statement and then it says you’ve got to talk to your GP, and I think the GP is your first port of call because that’s the authority you respect and you would take advice from, not a brochure.” Male
15	“I was just going to say if I was looking at this and I was interested the next thing I would go and look at would be Dr Google and then start doing some online research.” Male
16	“So, if he [my GP] had of said to me, also take this and have a read, I probably would have been on it.” Male
17	“Oh, I see Cancer Council as being like it’s approved, it’s, you know I sort of follow their guideline is like - I don’t know if I follow their guidelines, but I pay attention to their guidelines. If the Cancer Council say it, then I go well that’s been checked, as opposed to a drug company saying – not that I distrust drug companies – but I have a great deal of trust in the Cancer Council.” Male
18	“So, because I take it every day, I thought oh well if I can get an ulcer, I’ll get an ulcer, it’s better than having a heart attack, but it’s – maybe that’s not correct. Maybe low dose aspirin doesn’t.” Male
19	“I’d certainly talk to the GP about it. As I say, I’ve been taking aspirin for years and years. When you look at the statistics here, in terms of bowel [colorectal] cancer, it’s something that personally I’ve been worried about. Because like (another consumer), I’ve got a very bad family history of bowel [colorectal] cancer. If I wasn’t taking aspirin, I would consider taking it for the statistics you’ve got here.” Male
20	“I mean do you guys, this is going to sound really stupid, but being a little bit older than me, do you find that there’s a stigma associated with bowel [colorectal] cancer from your generation that people don’t want to talk about butts and bowel [colorectal] cancer and other bits and pieces. Because I find that with my dad, as I said he’s diagnosed four weeks ago, I know about it, my mum knows about it and my sister knows about it. He’s embarrassed, ashamed, humiliated, horrified.” Male
Approaches to implementation	
21	“Because the practice nurse, as defined, is usually in another room doing blood tests, they’re busy people.” Male
22	“No, it’s good. The problem you’ve got with the GP office, everybody’s GP’s like mine, they’ve got a zillion of these things in their offices. The walls covered with bloody signs and racks and racks of these kinds of things.” Male
23	“Because I sit in a waiting room and I look at all the stuff that’s on the board.” Female
24	“I sort of feel like this is the sort of thing that your doctor should give you if you’re at that age and you’ve got other issues and whatever, that this is sort of something they might say, well you know have a look at this…But it is nice and simple, just the formula, the way that’s laid out. These are things you would discuss with your GP.” Female
25	“But anyway, so when you’ve got a good doctor after you find one, then that’s fine. But if you’re just going to a real general, general practitioner then you might say, yeah, well I came in for a cold, or came in for whatever, a cut or whatever. Yeah, yeah, I’ll read that later or something.” Male
26	“With my diabetes and stuff, oh prevention, blah, blah, blah. Same with my blood clotting disorder, take this, this, this and this. Only really when it’s discovered. It’s not like do you have this; do you have that? It’s only known things that I’ve got, not preventative things that you think I might have if that makes sense.” Female

### Results from clinicians: feedback on EFTs

#### Ease of comprehension and design aspects of EFTs

To improve the comprehension of the EFTs, clinicians suggested several changes. Collectively, the clinicians found that they spent too long trying to calculate the differences between people who had taken aspirin and people who had not and suggested the addition of an absolute difference to the EFTs (Table 3, No 1; Table 4, Clinician quotation 1).

Clinicians suggested relative risk reductions presented as percentages in addition to the absolute difference to the EFTs. Previous research suggests that relative risk reductions would cause an overestimation of the benefits and risks of taking aspirin, and therefore this recommendation was not included in the revised EFTs.^10,17,18^ Additionally, the EFTs were already dense with numbers, and evidence suggests excessive information makes it difficult to process and comprehend the data.^19^ As a result, the steering group decided not to add the percentages to the EFTs (Table 4, Quotation 2).

Clinicians recommended the use of arrows to signpost numerical differences and make the risk of bleeding from the stomach and gut more obvious by changing its colour (Table 3, No 2; Table 4, Quotations 3 and 4).

The EFT went through several iterations of colour changes. A pharmacist recommended changing the colour of the female EFT so that it was not pink for women and blue for males, “And I think it should be neutral colours” (Female, Pharmacist). As a result, the female EFT was changed to yellow. However, more clinicians thought that the colours made the EFTs easy to differentiate for males and females. As a result, we changed the colour of the female EFTs to a lilac colour (Table 3, No 3; Table 4, Quotation 5). After these changes, the colours and arrows received positive feedback from the clinicians. To make the EFTs more inclusive, the male and female icons on the EFTs were removed, and instead, the font size for the words “men” and “women” was increased (Table 3, No 4; Table 4, Quotation 6).

#### Adding mortality data

Many clinicians mentioned the utility of adding all-cause mortality figures in the EFTs. Clinicians wanted to understand the effect of aspirin on several diseases, as well as side effects, e.g. the risk of gastrointestinal bleeding, and how these risks and benefits contributed to overall differences in risk of death (Table 3, No 5; Table 4, Quotation 7).

#### Potential effects on decision-making about aspirin

The clinicians felt consistently positive about using the EFTs for explaining the benefits and harms of taking aspirin to their patients. Clinicians’ positive attitude towards the DA could potentially encourage them to discuss them with their patients (Table 4, Clinician quotations 8–10).

While most clinicians agreed that they would use the EFTs for patients who were interested in preventive health, some thought it was too complicated, especially for patients with several chronic illnesses and lower health literacy levels. Some felt the EFTs were busy and contained too much information. Therefore, the next iteration was simplified but with a consensus from the steering committee to balance simplicity with accurate information (Table 4, Quotation 11).

Clinicians agreed that because the EFTs showed that the benefits outweighed the risks of taking aspirin, they would prescribe aspirin to their patients. They also mentioned that the side effects, although unpleasant, were better than being diagnosed with cancer (Table 4, Quotation 12). In contrast, some clinicians simply thought that their patients would not be interested in taking aspirin and much of their patient population would not find the EFTs to be useful (Table 4, Quotation 13).

#### Approaches to implementation

Clinicians discussed barriers and facilitators to implementing the EFT into clinical practice and gave suggestions for implementing it into clinical practice. Barriers included a lack of time (Table 4, Quotation 14). They recognized that the general practice setting was best suited to implement the EFT, as GPs already spend time on preventative health and managing side effects from medications (Table 4, Quotation 15). Clinicians also suggested that the EFT could be disseminated to GPs through the Royal Australian College of General Practitioners (RACGP), similar to the DA for prostate cancer screening (Table 4, Quotation 16).

### Results from consumers: feedback on EFTs and DAs

#### Ease of comprehension and design aspects

Consumers’ first impressions of the DA were positive. They liked that the DA was concise and clearly communicated the guidelines. The first, second, and final progressive iterations of the DA before, during and after consumer feedback can be found in Supplementary Files D–H. Consumers generally thought the dose of aspirin, they were recommended based on the DA was ambiguous (100–300 mg) and being given a range made it difficult to understand. Four of the consumers were currently taking low-dose aspirin (100 mg), so they found the range particularly confusing (Table 2).

Consumers were also confused about how long they should take aspirin after looking at the DAs. (Table 5, Quotations 1 and 2). To minimize confusion, they recommended that the information about the dose and time were split and wording altered (Table 3, No 6; Table 5, Quotations 3 and 4). Consumers also generally did not know what the contraindication, “*H. Pylori*” meant and suggested removing it from the DA (Table 5, Quotations 5 and 6). However, the steering committee decided against removing *H. pylori* from the DA, as it is a significant contraindication to taking aspirin. Additionally, the DA was designed to be used with a clinician who will be able to explain *H. pylori* to the patient and may require prompting about this contraindication.

Consumers suggested it was not immediately clear which DA was for males or females by the front page; this resulted in us adding “man” and “women” on the front in a larger and bolded font. Consumers also suggested that we combine the 2 DAs into one, but the steering committee decided against this, as the DA would then be overcrowded with two EFTs (Table 3, No 8; Table 5, Quotation 7).

Consumers also felt that the pink female DA with the image of a woman with close-cropped hair on the front looked like a breast cancer survivor and may incorrectly lead people to think that the brochure is associated with breast cancer awareness. This aligned with the clinicians’ proposal that a different colour other than pink should be used. Ultimately, the colour was changed to lilac, and an image of a woman with long hair (Table 3, No 7; Table 5, Quotation 8).

Finally, consumers found the font difficult to read, therefore the font size was made larger throughout the DA. Consumers also did not think the tablet image in the second iteration of the DAs (Supplementary Files E and F) looked like aspirin, so we changed the image of the large tablet to a blister packet of aspirin tablets (Supplementary Files G and H; Table 3, No 9 and 10; Table 5, Quotations 9–11). Consumers were posted the final version of the DAs and no further suggestions for change were provided.

### Potential effects on decision-making about aspirin for consumers

Consumers discussed how they would come to a decision on whether to take aspirin after being shown the DAs. They compared them to other GP waiting room brochures and thought they were better. They felt that the statistics presented in the EFTs could encourage them to take aspirin (Table 5, Quotations 12 and 13).

Consumers discussed how they would come to the decision to take aspirin. They recognized the need to speak to their GP about taking aspirin and also said that they would Google or search for evidence on their own, in addition (Table 5, Quotations 14 and 15). As the consumers generally trusted their GPs, if the GP recommended they take aspirin, they would oblige (Table 5, Quotation 16). Consumers noticed the Cancer Council Australia logo on the DAs, and as a trusted organization, they felt reassured that the guidelines were not published by a drug company. (Table 5, Quotation 17).

After seeing the DAs, consumers recognized that the benefits of aspirin outweighed, especially when weighed against the alternative, that is, bowel cancer, even if they experienced side effects from aspirin. The EFT statistics were also powerful in convincing consumers that taking aspirin was worthwhile as they saw the difference in numbers between those who took aspirin versus those who did not take aspirin as large enough to take it (Table 5, Quotations 18 and 19). There was also some perceived stigma about colorectal cancer and although the DAs are about prevention, their focus on colorectal cancer made consumers think that people might not want to look at the DAs (Table 5, Quotation 20).

### Approaches to implementation

Consumers offered suggestions for how the DAs could be implemented. Barriers to implementing the DAs included a perception that clinicians would be too busy to discuss the DAs with patients (Table 5, Quotation 21).

During the focus groups, although it was mentioned that the DA would be used in consultation with a clinician, the consumers associated the format of the DA with brochures they often see in general practice waiting rooms. Consumers cautioned against making the DAs available in general practice waiting rooms, as there are already too many, and people tended to be ignored (Table 5, Quotations 22 and 23).

Consumers agreed that GPs should provide the DAs when patients reach 50 years old. They agreed that their GPs were best suited to provide information on the DA as they already know their health history and usually have ongoing relationships with patients (Table 5, Quotation 24).

Consumers recognized the need for DAs to be available from pharmacists after one consumer said, “Yeah, but somebody like me who doesn’t go to the doctor very often, I’m not likely to see it I don’t think.” Male

Consumers mentioned the importance of having proactive GPs to support the guidelines. They suggested that if GPs were “good” then they would know about the DAs and share them with their patients. In contrast, if GPs did not have an interest in preventative medicine, they may not use them. Some consumers reported their GPs do not discuss preventative health interventions; rather, their GPs tend to focus on the reason for the consultation, such as chronic diseases, thus preventive care is not addressed (Table 5, Quotations 25 and 26).

## Discussion

It is projected that there will be a 59.2% increase in colorectal cancer mortality in Australia between 2013 and 2035, the second highest in the world.^20^ To our knowledge, this is the first Australian study to iteratively develop and design DAs about the benefits and risks of taking aspirin to prevent colorectal cancer and other chronic illnesses using feedback from clinicians and consumers. Our study shows feedback from both clinicians and consumers enhanced the design and eased the comprehension of the EFTs and DAs. This could, as a result, assist in decision-making regarding aspirin consumption, and may encourage consumers to speak to their GP before taking aspirin. Consumers and clinicians also shared ideas for the implementation and dissemination of the DAs.

Clinician and consumer feedback on the EFTs and DA, respectively, were invaluable for improving comprehension and optimizing the design of a DA. There have been several studies where DAs have been developed with input from consumers and clinicians, which improved their comprehension.^7,21,22^ McDonnell’s study of a lung cancer screening DA for use in primary care incorporated feedback from 20 patients in an iterative process and resulted in the use of more plain language and showed that DAs can be used to educate both clinicians and patients about new guidelines.^22^ In our study, both the EFTs and a DA changed in both language and design, which could also impact the decision on whether to take aspirin.

We made further changes that were consistent with current evidence for designing effective DAs. Incoporating numerical data was vital for our DA, and we utilized the EFT to effectively convey the importance of decision making while considering the statistics.^23^ However, the evidence also suggests a DA must include more than just numerical information as people understand risk in different ways.^7^ Our DA also included a written recommendation, a reminder to screen for colorectal cancer and contraindications to take aspirin.

In the current study, consumers changed the wording throughout the DA to make it comprehensible, although we left some of the original wording to not compromise on clinical accuracy and to prompt clinicians. Clinicians mentioned that they wanted percentages showing relative risk reductions to be added to the EFTs, but we decided it was best not to add them as international guidelines for DAs show that the use of absolute risks and frequencies rather than risk ratios lead to people the overestimating size of benefits and harms.^17,24^

Clinicians and consumers thought the EFTs and DAs presented the harms and benefits of taking aspirin clearly, which could help consumers make a better-informed decision on whether to take aspirin. Dawn and colleagues’ systematic review found that DAs improved patients’ knowledge as well as supported and increased informed choices when patients were faced with screening or health treatment decisions.^7^ Qualitative data from this study show that consumers and clinicians believe that our EFTs and DAs could positively impact informed decision-making.

If a DA is used in consultation with a clinician, it can improve knowledge regarding the benefits and harms of an intervention, as found by Reuland and colleagues in their study of a lung cancer screening DA used in primary care.^25^ We designed our DAs to be used in consultation with a clinician and after consumers were shown the DAs in the focus groups, they agreed that it would encourage them to have a discussion with their GP as they recognized the need to discuss the benefits and contraindications before commencing aspirin.

Although long-term use of low-dose aspirin has similar relative benefits and risks for men and women, the absolute benefits in terms of colorectal cancer and cardiovascular disease reduction are greater for men. Clinicians and consumers also mentioned that a potential barrier to implementing the DAs was having separate versions for men and women and suggested that we combine them to form one. The steering group decided against combining the DAs as it is important for males and females to know that the absolute risk benefits are different and it is common for sex-specific differences to be shown separately on DAs.^26,27^

To strike a careful balance between including too much information in one DA and presenting accurate information, it was decided not to combine the male and female DA into one brochure. The consumers in our study appreciated that the DAs did not contain too much information in comparison to other brochures they had seen. In one study of a DA for women facing breast cancer surgery, women viewing the DAs sometimes did not finish reading them, and some chose not to read them at all because they contained too much information.^28^ In another study, 87% of clinicians reported a need for patient educational materials about aspirin to prevent colorectal cancer for high-risk patients.^29^ After ranking what types of educational materials clinicians preferred out of 6 types, they ranked a brief informational brochure as the most preferred, and a paper DA as the second most preferred.

In a recent study, researchers from the United Kingdom^26^ designed a DA based on both the Australian aspirin chemoprevention guidelines and the United States Preventive Services Task Force (USPTF)^30^ aspirin guidelines with feedback from clinicians (*n* = 10) and consumers (*n* = 14). They found that their DAs were acceptable and could support decision-making. Their participants saw an opportunity to include GPs and pharmacists in the decision-making process. In our previous work, we found clinicians agreed that the DAs should be implemented through general practice.^5^ Our DAs contrast heavily in design as well, with our DAs including far less text and a visual representation of the benefits and risks of the EFT. This study solely covers the development of the DA which is currently undergoing further testing of its impact on the uptake of aspirin and informed decision-making about aspirin in general practice as a part of an RCT—the “Should I Take Aspirin” (SITA) trial.^31^

### Implications and limitations

This study has resulted in well-refined comprehensible and clearly designed DAs that present the aspirin guidelines for use in consultation with a clinician. As a part of this study, we interviewed different clinician specialties, many with varying years of experience, and ages. The diversity resulted in a comprehensive review of the EFTs. We also included feedback from consumers in focus groups, which further added to the refinement of the DAs.

There are some limitations of our study; firstly, only a small number of consumers participated in the focus groups, and only two focus groups were conducted, due to the COVID-19 pandemic. Another limitation is that we recruited clinicians and consumers only from Victoria, who only spoke English and identified as male or female and were mainly white and educated. We did not include any Aboriginal or Torres Strait Islander clinicians or consumers. We did include consumers on the wider project steering committee.

Despite these limitations, we sent the final versions of the DAs to the consumer participants, which they approved. Our study population of clinicians and consumers were from regional and metropolitan locations, representing the views of many Australians.

## Conclusion

This study has produced an intervention in the form of a DA that may be effective for raising awareness of the guidelines and supporting informed choice about taking aspirin to potentially increase aspirin uptake. The design and comprehension of the DAs were improved through clinician and consumer input. The final versions of the DAs are being used in general practice as part of the SITA trial, which aims to increase aspirin uptake and support patients’ informed choices.^31^ Pending the results of the trial, we plan to implement the DAs in general practice and make them publicly available for clinicians to use. This could be supplemented by approaches to raise awareness in the community about the role of aspirin and the tools to facilitate discussions between GPs and patients to support informed choices about colorectal cancer prevention.

## Supplementary material

Supplementary material is available at *Family Practice* online.

cmad042_suppl_Supplementary_Files

## Data Availability

Please contact SM if you would like to access any data.
