# Supplementary material for: Designing a decision aid for cancer prevention: a qualitative study
Source: Fam Pract. 2023 Apr 14;41(3):349–59. doi: 10.1093/fampra/cmad042 (PMC11167968; doi:10.1093/fampra/cmad042)
Supplement: cmad042_suppl_Supplementary_Files [file cmad042_suppl_supplementary_files.docx]

Supplementary Files for: Designing a decision aid for cancer prevention

#### *Supplementary file A. Interview schedule for clinicians, extracted questions for this project.*

**INTERVIEW SCHEDULE FOR CLINCIANS**

Project: What are clinicians’ views and opinions about prescribing aspirin to reduce the risk of developing colorectal cancer?

Interviews will be guided by the following schedule which only provides general areas to be covered.

**INTRODUCE CANCER COUNCIL GUIDELINES**
***(Show laminated version of summary / recommendations)*** *(“So I have a summary of the guidelines, because we are recording it would be great if you could ‘think out-loud’ as you review it”)*

- Are you aware of the new guidelines? What is your understanding of the aspirin recommendations?
- Are you aware of guidelines that recommend prescribing aspirin to prevent bowel cancer?

**INTRODUCE** **EXPECTED FREQUENCY TREES**

*Show clinician the expected frequency trees –* ***incidence*** *and* ***mortality****. (“again, could you please think out loud as you review it”) Provide* ***evidence*** *for where the* ***numbers come from.*** *Emphasise it was developed for people aged* ***50-70.***

- What do you think about the expected frequency tree?
- Would the decision aid be helpful in these discussions with pts?

Supplementary file B. Focus group interview schedule.

**INTERVIEW SCHEDULE FOR FOCUS GROUP**

Project: What are consumers’ views and opinions about prescribing aspirin to reduce the risk of developing colorectal cancer?

****Remind them that you’ll be recording the interview, have everyone introduce themselves, say name**

**CHECK AWARENESS**

- Are you aware of that aspirin can be taken to reduce your bowel [colorectal] cancer risk?
  - Is yes from anyone, what is your understanding of the aspirin recommendations?
- Are you aware that Australian guidelines recommend that GPs should discuss aspirin to reduce your risk of bowel [colorectal] cancer?

**OPINION ON RECOMMENDATIONS**

- What do you think aspirin might be used for?
  - Some people might just bring up here that they are taking aspirin. If so, and they are happy to elaborate, explore why they are taking it.
- What do you think about using aspirin to prevent bowel cancer?
- Do you know any side effects of aspirin and if so, what are they?

***No questions here…INTRODUCE*** ***DECISION AID***
*Show participants the* ***2*** *decision aids****.***

- Let them know that the guidelines were published through the Cancer Council Australia in 2017 but there was no plan to implement them.
- (Give them 3-5 minutes to read through the decision aid, gauge the room and look for everyone to be done)

**OPINION AND COMPREHENSION**

- What do you think of the decision aids? What do you think of the colours?
- What do you think of the figure show the effects of aspirin? Can you understand it?
- Are there any points that need to be clarified?
- How might you respond if a practice nurse discussed this brochure with you? Would it prompt you to speak to you GP about taking aspirin?
  - Would you start taking aspirin on your own without consulting your GP?
- Would you make any changes? Is there anything specifically that you don’t like/understand

#### *Supplementary file C. Methods for calculating the final all-cause mortality estimates in Expected Frequency Trees*

The methods used to calculate the absolute numbers of clinical outcomes for the first version of the EFTs are described elsewhere.^1^ These data are from two meta-analyses of large randomised controlled trials.^2,3^ Subsequent versions of the EFTs were updated to incorporate competing risks of mortality into the incidence figures. Australian age- and sex-specific all-cause mortality rates^4^ were used to calculate the probability of survival over the next 10 years, then these figures were incorporated within calculations of 10-year absolute risks of each clinical outcome with and without aspirin use for five years. Specifically, baseline incidences of colorectal cancer^5^, heart attack^6^ and stroke^7^ and GI bleeding^8^ were converted to absolute 10-year risks, accounting for competing risks of mortality, and then absolute or relative risk differences with aspirin use were applied (for heart attacks, strokes, and major GI bleeds, using figures from Cuzick et al.^8^, and for colorectal cancer, using figures from Rothwell et al^3^). Absolute numbers of events over ten years were then calculated using Australian age- and sex-specific population figures^9^.

Initial feedback from clinicians suggested utility in providing absolute differences in mortality for those taking aspirin compared to those who did not. Estimates for all-cause mortality per 10,000 Australian men and women were calculated. The Australian age- and sex-specific mortality rates^4^ were converted to a 10-year cumulative risk of mortality as a baseline. The 10-year risk of mortality for those who take aspirin for five years was then calculated using absolute risk reductions reported by Cuzick et al^8^. Australian age- and sex-specific population figures^9^ were then applied to these absolute risks to determine absolute all-cause mortality numbers over ten years.

### *References*

1. Nguyen P, McIntosh J, Bickerstaffe A, Maddumarachchi S, Cummings KL, Emery JD. Benefits and harms of aspirin to reduce colorectal cancer risk: A cross-sectional study of methods to communicate risk in primary care. *Br J Gen Pract*. 2019;69(689):E843-E849. doi:10.3399/bjgp19X706613

2. Rothwell PM, Fowkes FGR, Belch JF, Ogawa H, Warlow CP, Meade TW. Effect of daily aspirin on long-term risk of death due to cancer: analysis of individual patient data from randomised trials. *Lancet*. 2011;377(9759):31-41. doi:10.1016/S0140-6736(10)62110-1

3. Rothwell PM, Wilson M, Elwin C-E, et al. Long-term effect of aspirin on colorectal cancer incidence and mortality: 20-year follow-up of five randomised trials. *Lancet*. 2010;376(9754):1741-1750. doi:10.1016/S0140-6736(10)61543-7

4. General Record of Incidence of Mortality (GRIM) books, Data visualisation - Australian Institute of Health and Welfare. Accessed January 28, 2022. https://www.aihw.gov.au/reports/life-expectancy-death/grim-books/contents/general-record-of-incidence-of-mortality-grim-books

5. Colorectal and other digestive-tract cancers, Summary - Australian Institute of Health and Welfare. Accessed February 28, 2022. https://www.aihw.gov.au/reports/cancer/colorectal-other-digestive-tract-cancers/summary

6. Health - Report on Government Services Productivity Commission. Accessed February 28, 2022. https://www.pc.gov.au/research/ongoing/report-on-government-services/2018/health

7. https://www.aihw.gov.au/getmedia/4a758db2-37c6-49b69f9e-578213fb42ed/aihw-cvd-data-tables-2017.xls.aspx. Accessed February 28, 2022.

8. Cuzick J, Thorat MA, Bosetti C, et al. Estimates of benefits and harms of prophylactic use of aspirin in the general population. *Ann Oncol*. 2015;26(1):47-57. doi:10.1093/annonc/mdu225

9. Population Projections, Australia, 2017 (base) - 2066 | Australian Bureau of Statistics. Accessed January 28, 2022. https://www.abs.gov.au/statistics/people/population/population-projections-australia/latest-release


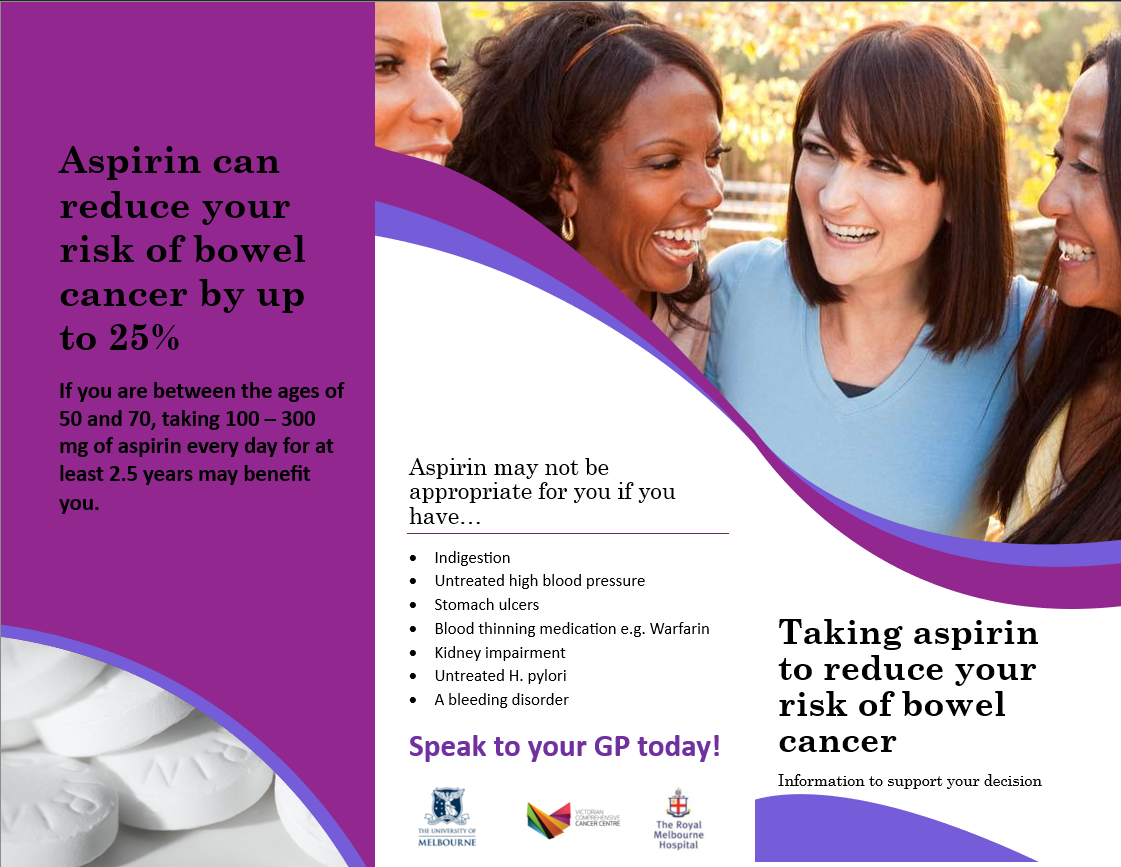


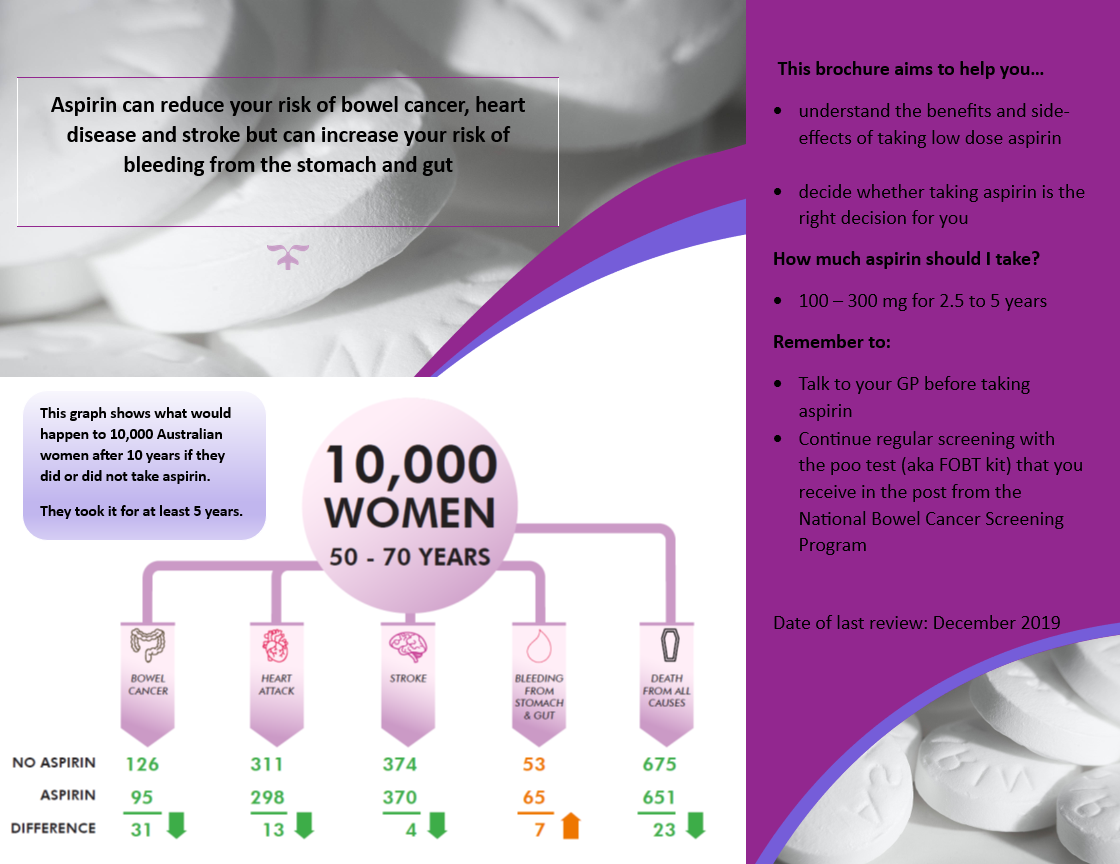
Supplementary file D. First iteration of the tri-fold female decision aid before consumer focus group feedback 1st draft, including only feedback from the steering group members.


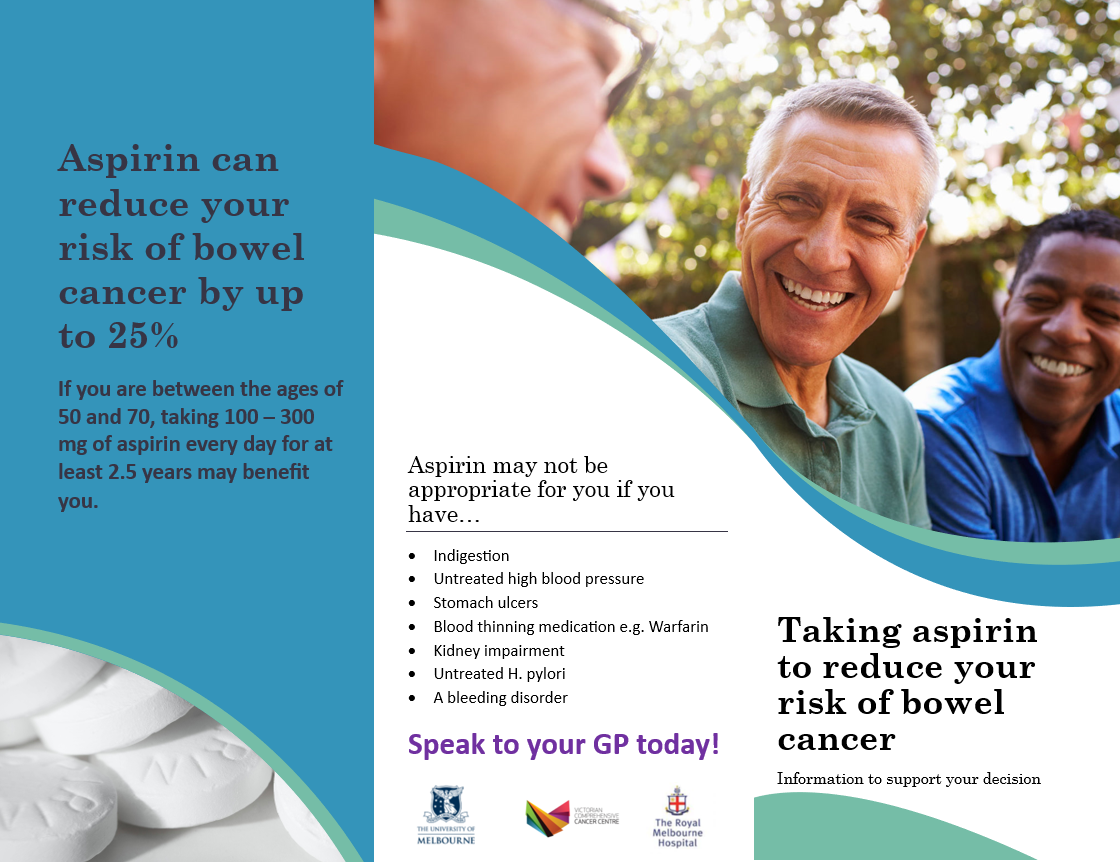


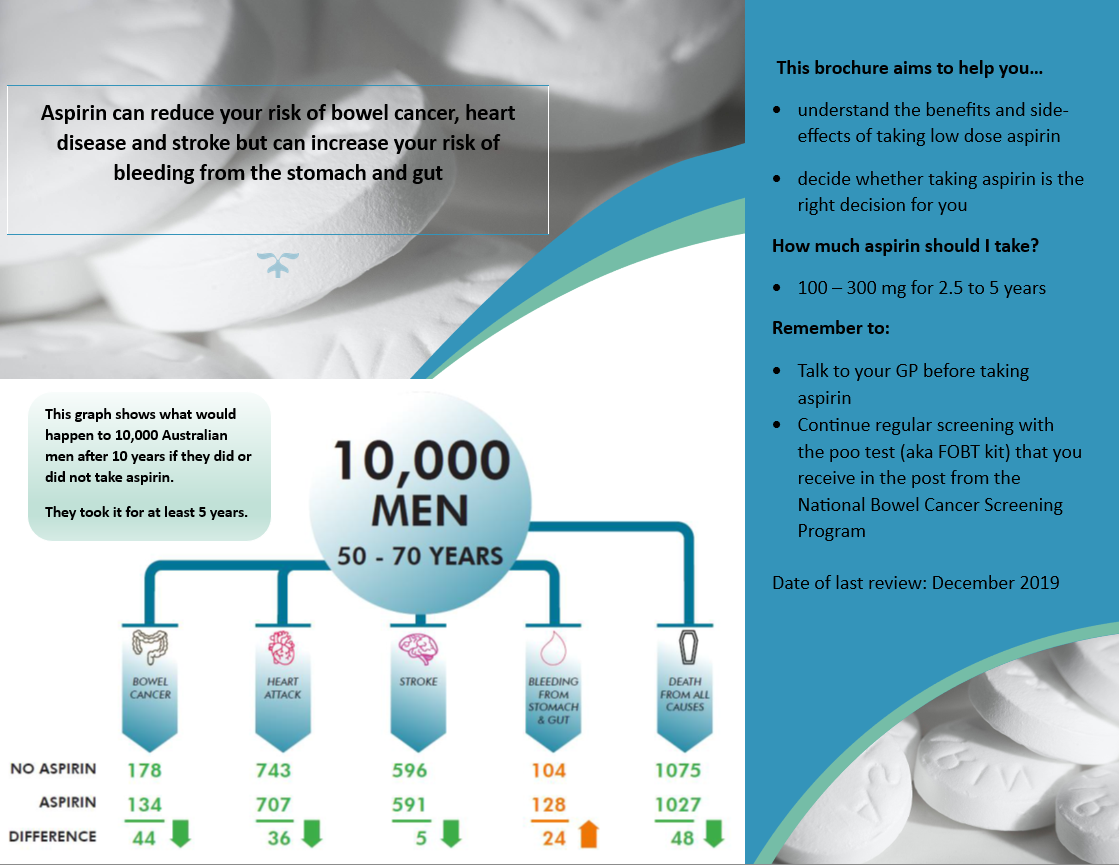

Supplementary file E. First iteration of the tri-fold male decision aid before consumer focus group feedback, including only feedback from the steering group members.


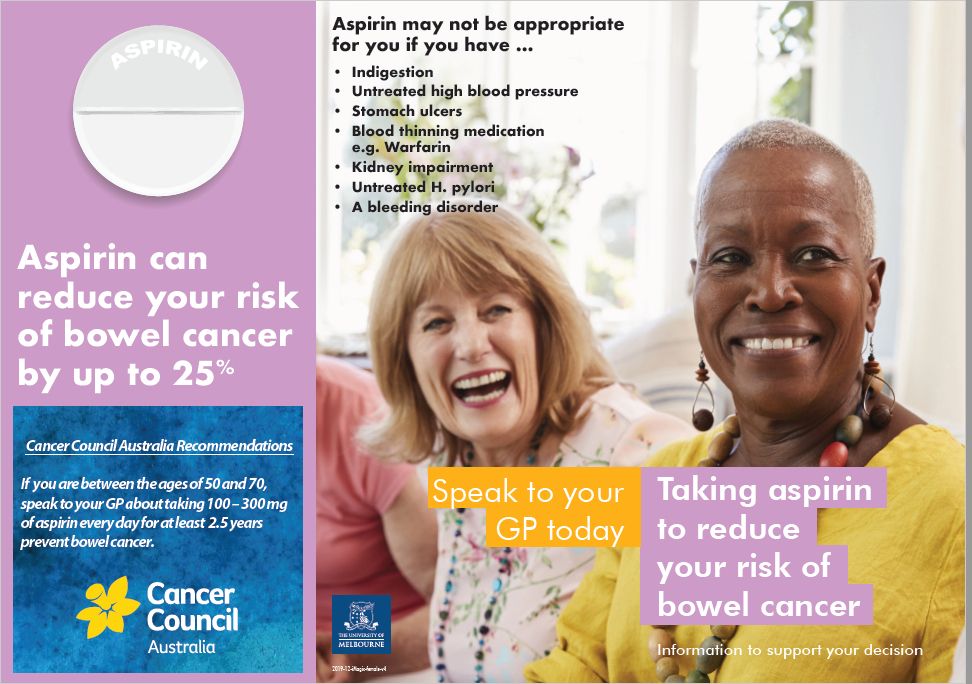


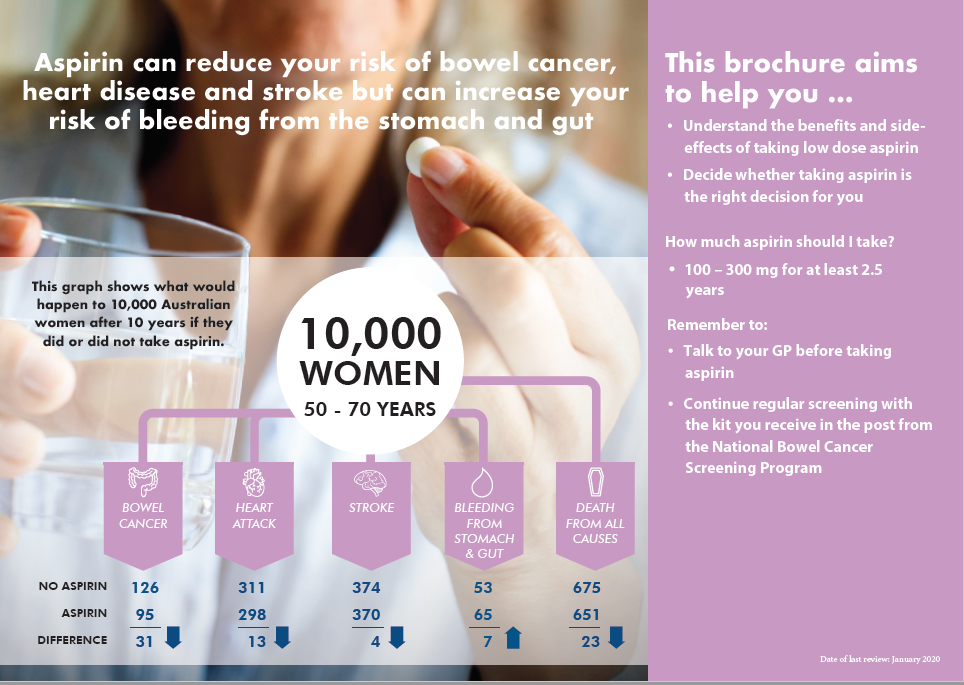


### Supplementary file F. Second iteration of the tri-fold female decision aid before consumer focus group feedback.


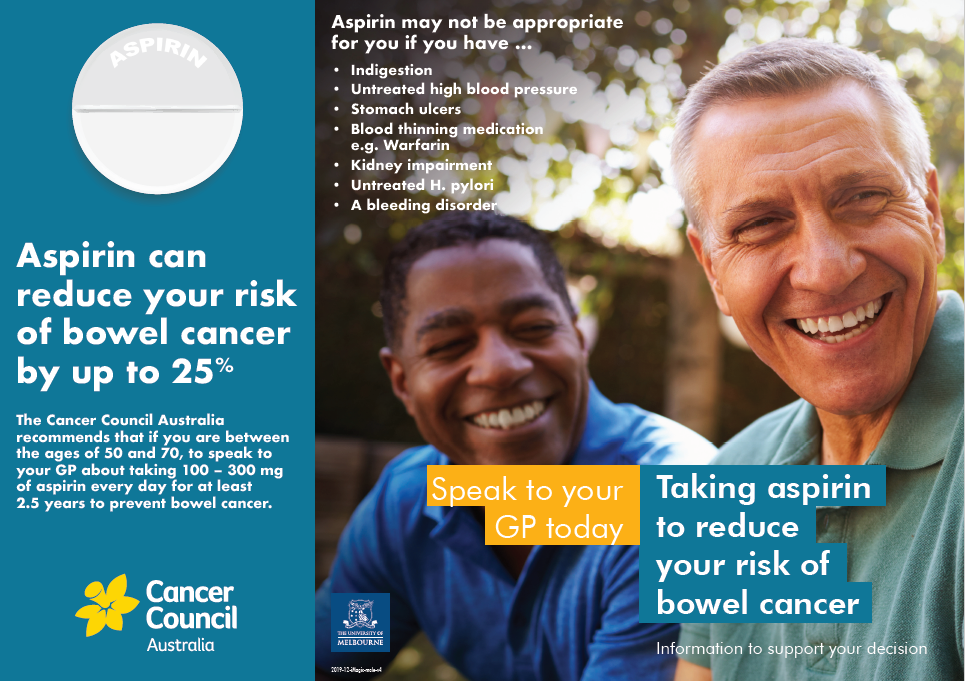


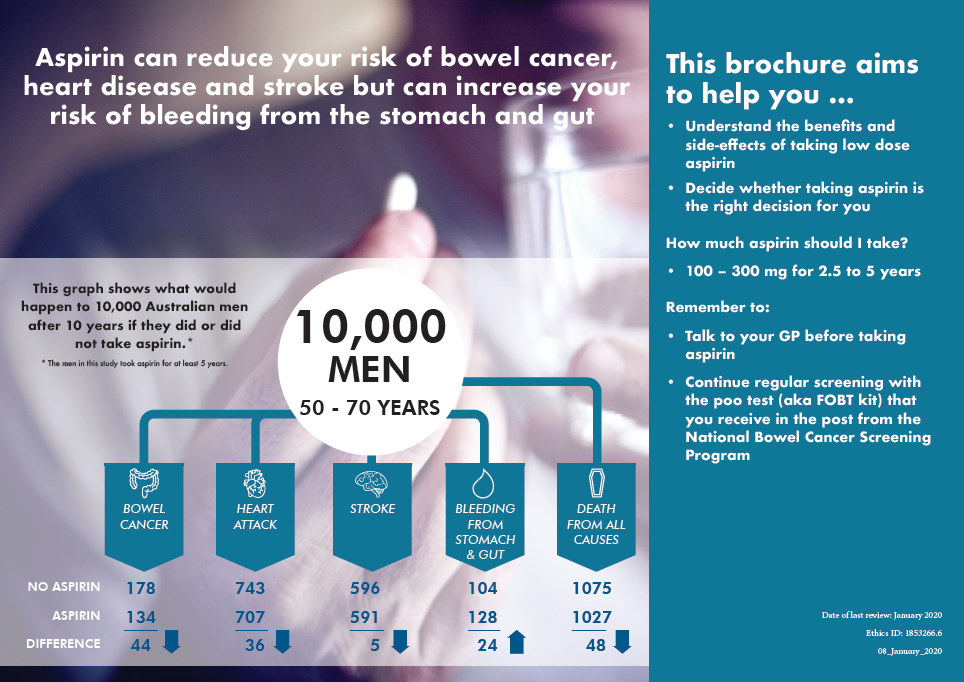


### Supplementary file G. Second iteration of the tri-fold male decision aid before consumer focus group feedback.


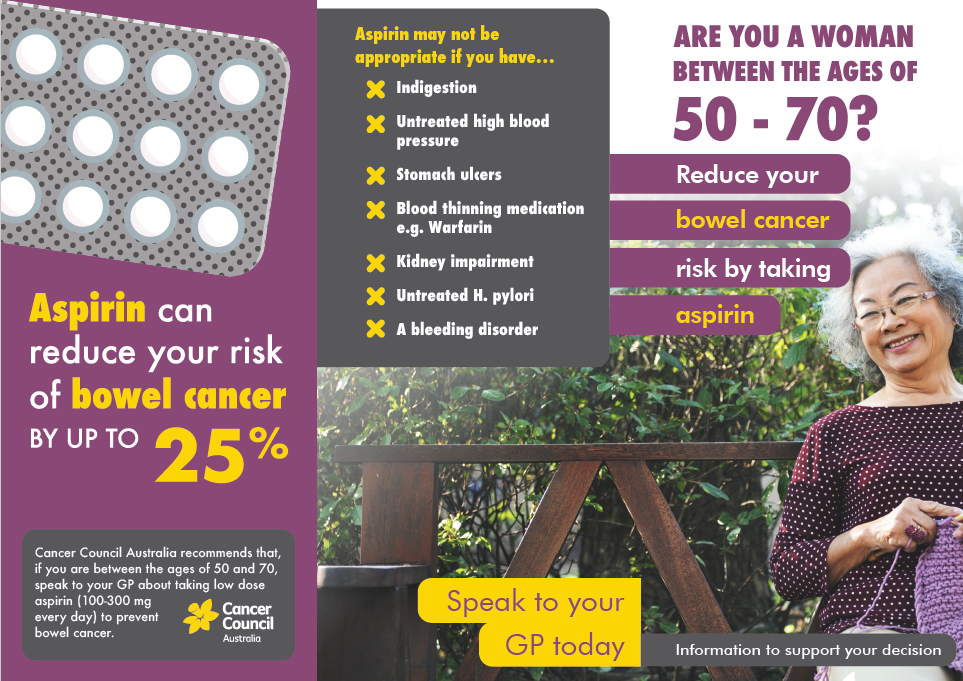


###
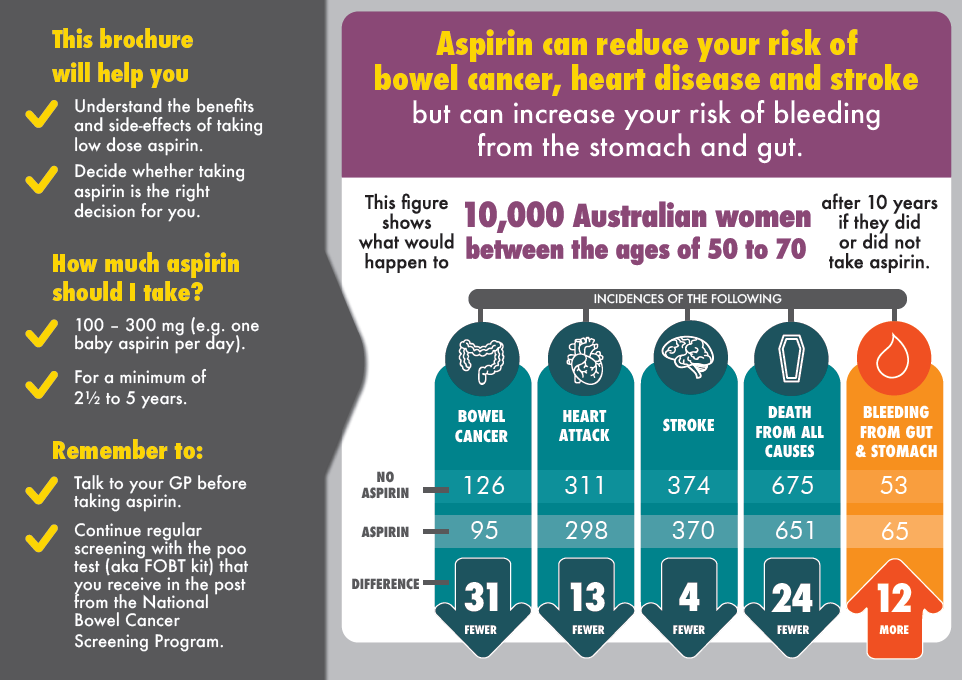
 Supplementary file H. Final version of the tri-fold female decision aid after incorporating results from focus groups.


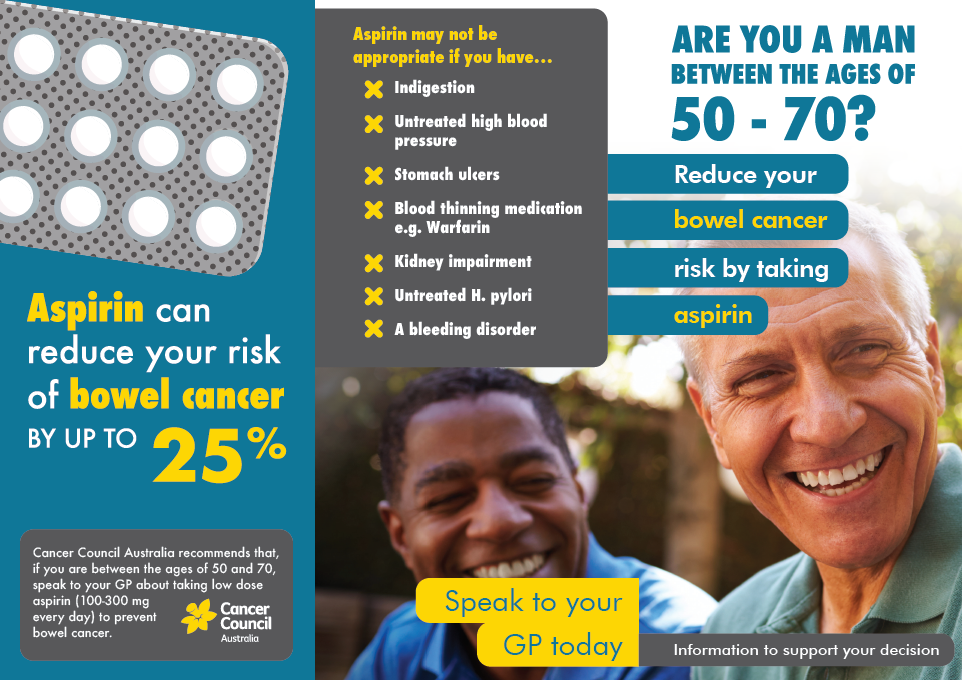


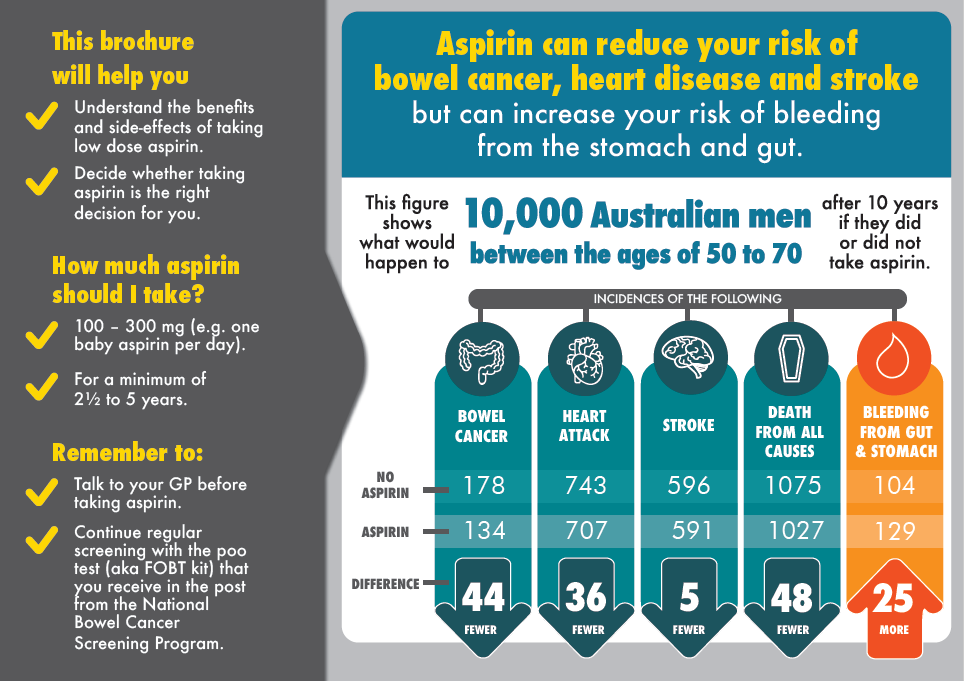
Supplementary file I. Final version of the tri-fold male decision aid after incorporating results from focus group.
